# Screening method to identify hydrogel formulations that facilitate myotube formation from encapsulated primary myoblasts

**DOI:** 10.1002/btm2.10181

**Published:** 2020-09-03

**Authors:** Dhananjay V. Deshmukh, Nils Pasquero, Gajraj Rathore, Joel Zvick, Ori Bar‐Nur, Jurg Dual, Mark W. Tibbitt

**Affiliations:** ^1^ Macromolecular Engineering Laboratory, Department of Mechanical and Process Engineering ETH Zurich Zurich Switzerland; ^2^ Institue for Mechanical Systems, Department of Mechanical and Process Engineering ETH Zurich Zurich Switzerland; ^3^ Laboratory of Regenerative and Movement Biology, Department of Health Sciences and Technology ETH Zurich Schwerzenbach Switzerland

**Keywords:** 3D culture, immunostaining, muscle cells, parametric analysis, primary murine myoblasts, rapid screening

## Abstract

Hydrogel‐based three‐dimensional (3D) cellular models are attractive for bioengineering and pharmaceutical development as they can more closely resemble the cellular function of native tissue outside of the body. In general, these models are composed of tissue specific cells embedded within a support material, such as a hydrogel. As hydrogel properties directly affect cell function, hydrogel composition is often tailored to the cell type(s) of interest and the functional objective of the model. Here, we develop a parametric analysis and screening method to identify suitable encapsulation conditions for the formation of myotubes from primary murine myoblasts in methacryloyl gelatin (GelMA) hydrogels. The effect of the matrix properties on the myotube formation was investigated by varying GelMA weight percent (wt%, which controls gel modulus), cell density, and Matrigel concentration. Contractile myotubes form via myoblast fusion and are characterized by myosin heavy chain (MyHC) expression. To efficiently screen the gel formulations, we developed a fluorescence‐based plate reader assay to quantify MyHC staining in the gel samples, as a metric of myotube formation. We observed that lower GelMA wt% resulted in increased MyHC staining (myotube formation). The cell density did not significantly affect MyHC staining, while the inclusion of Matrigel increased MyHC staining, however, a concentration dependent effect was not observed. These findings were supported by the observation of spontaneously contracting myotubes in samples selected in the initial screen. This work provides a method to rapidly screen hydrogel formulations for the development of 3D cellular models and provides specific guidance on the formulation of gels for myotube formation from primary murine myoblasts in 3D.

## INTRODUCTION

Three‐dimensional (3D) cellular models are increasingly applied to study cell and tissue biology or pathology outside of the body as well as to test potential therapeutic modalities.^1^ 3D cellular models are often composed of one or more cell types embedded within a synthetic or natural hydrogel that is designed to mimic relevant aspects of the extracellular matrix (ECM).^2^, ^3^ Synthetic or semisynthetic hydrogels are particularly attractive as they can be designed with similar physical and biochemical properties as the ECM, while providing a reproducible scaffold to investigate physiology ex vivo.^4^ However, the properties of a hydrogel scaffold can influence different cell types in unique ways. For example, soft and macroporous hydrogels have been designed for neuronal cell growth and enzyme‐degradable hydrogels have been engineered for intestinal organoid culture.^5^, ^6^ Therefore, hydrogel compositions are often tailored to the cell or tissue type and for the specific biological goal of the 3D model.

Skeletal muscle is one specific tissue of interest for the design of 3D cellular models, which can be used to study myogenesis and investigate the pathophysiology of common muscle disorders.^7^ Skeletal muscle is comprised of contractile myofibers and innervating motor neurons embedded within a surrounding vascularized matrix composed of connective tissue cells. During muscle development and repair, myogenic progenitor cells differentiate to myoblasts, which fuse to form myotubes, further maturing into contractile myofibers.^8^ Myoblasts can be sourced using the C2C12 cell line or through the isolation and culture of primary myoblasts from animal or human tissue.^9^, ^10^ C2C12 is an immortalized cell line that shows lower expression of muscle specific markers as compared with primary myoblasts.^11^ One critical requirement for these models is the functional fusion of myoblasts into contractile myotubes, which comprise the base unit of skeletal muscle and are useful for studying myogenesis and muscle biology ex vivo. Researchers have encapsulated myoblasts in a range of natural, synthetic, or semisynthetic hydrogels, including collagen, fibrin, Matrigel, gelatin, alginate, and poly(ethylene glycol) (PEG)‐based gels. In these experiments, it was found that several matrix parameters, such as gel stiffness, cell density, and molecular effectors, such as matrix proteins or growth factors, can influence myoblast proliferation as well as myotube formation and function.^12^ C2C12 myoblasts proliferation increased in alginate gels with low degradability; however, myotube fusion was favored in gels with high degradability.^13^ Cell density influenced contractile force generation of primary murine myoblasts in collagen gels; increased contractile force was observed with 6 × 10^6^ cells ml^−1^ as compared with 4 or 8 × 10^6^ cell ml^−1^.^14^ In another work, contractile muscle tissue models were bioprinted using myogenic progenitor cells (~5 × 10^6^ cells ml^−1^) in methacryloyl gelatin (GelMA).^15^ During 2D culture, primary myoblasts are often cultured on Matrigel‐coated tissue‐culture polystyrene, as Matrigel facilitates myoblast adhesion.^16^ To improve myoblast function in 3D, researchers have also encapsulated primary human skeletal muscle cells in hydrogels containing a mixture of collagen and Matrigel.^17^ Together, these studies illustrate that primary myogenic cells, or myogenic cell lines, can be encapsulated within hydrogel materials to develop 3D models of skeletal muscle; however, standardized conditions do not exist for developing 3D models of skeletal myotubes.

Generally, the selection of hydrogel compositions for 3D cellular models, such as a model of skeletal myotube formation and function, is carried out through a series of iterative encapsulation experiments, based on known hydrogel materials and guidance from the literature. While this trial‐and‐error approach is often able to identify suitable material compositions, the process can be time consuming and may overlook suitable mechanical properties and biochemical cues beneficial to the specific cell of interest.

**FIGURE 1:**
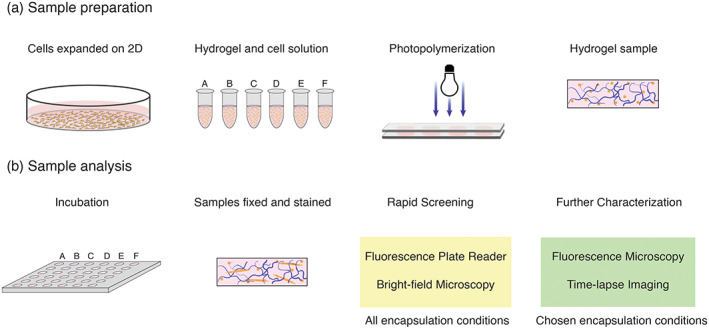
Screening encapsulation conditions for myotube formation from primary murine myoblasts in GelMA hydrogels. (a) Primary murine myoblasts were expanded on Matrigel‐coated petri dishes, trypsinized, and re‐suspended to prepare the gel formulations for the screening experiment. The myoblasts were encapsulated within GelMA hydrogel disks (10 μl; 1 mm thick) of the specified formulation using photopolymerization (*λ* = 365 nm; *I* = 20 mW cm^−2^; *t* = 30 s). (b) The samples were incubated in growth medium for 3 days, transferred to differentiation medium to induce myogenesis, and cultured for an additional 7 days. To perform the screening experiment, samples were then fixed and stained for myosin heavy chain (MyHC), as a marker of myotube formation. A rapid screen was developed using a fluorescence‐based plate reader assay and bright‐field microscopy for all conditions. Further characterization was carried out using fluorescence microscopy and time‐lapse imaging

In this work, we applied a parametric analysis to identify appropriate hydrogel compositions for myotube formation from primary murine myoblasts in 3D GelMA hydrogels (Figure 1). In particular, we investigated the effect of GelMA weight percent (wt%), cell density, and Matrigel concentration on myotube formation. This was identified by myosin heavy chain (MyHC) immunocytochemistry, 7 days after initiating differentiation. To improve the throughput of the parametric analysis, we developed a fluorescence‐based assay using a multi‐well plate reader to rapidly quantify MyHC expression in the different gel formulations.

The rapid screen refined the material compositions that facilitated myotube formation, which were confirmed by fluorescence microscopy and optical imaging of spontaneously contracting myotubes. We observed that increasing GelMA wt% had the strongest inhibitory effect on myotube formation; that the presence of Matrigel improved myotube formation as compared with pure GelMA samples; and that cell density did not significantly affect myotube formation. In total, the combination of design of experiments using parametric analysis and facile plate reader‐based assays offers an attractive approach to screen for hydrogel compositions that are suitable for engineering 3D cellular models.

## RESULTS AND DISCUSSION

In preliminary experiments to form myotubes in 3D hydrogel culture, we encapsulated primary murine myoblasts in GelMA and matrix metalloproteinase (MMP) degradable PEG‐based hydrogels (Figure S1). We observed that myoblasts proliferated more in GelMA compared with PEG‐MMP gels. Limited myotube formation occurred in GelMA hydrogels, and myotube formation increased by supplementing GelMA hydrogels with 1.2 mg ml^−1^ Matrigel (Figure S1). To determine if hydrogel formulation could be used to increase the extent of myotube formation, a parametric analysis was designed to screen the effect of GelMA hydrogel formulation on myotube formation (Figure 1).

**FIGURE 2:**
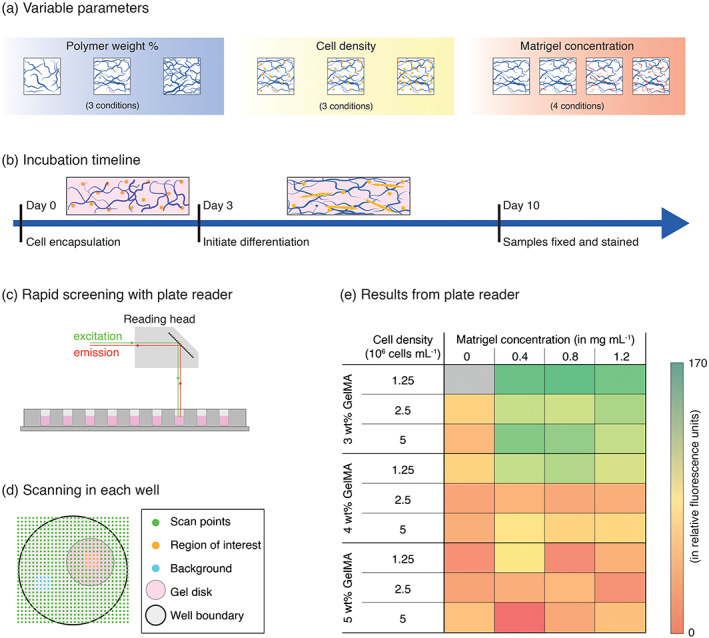
MyHC immunostaining and plate reader‐based screening of encapsulation conditions. (a) We prepared hydrogel samples for the encapsulation of primary murine myoblasts varying GelMA weight percent (3, 4, and 5 wt%), cell density (1.25, 2.5, and 5 × 10^6^ cells ml^−1^), and Matrigel concentration (0, 0.4, 0.8, and 1.2 mg ml^−1^). (b) All samples were prepared on Day 0 and cultured in growth medium for 3 days. Differentiation was initiated by transferring the samples to low‐serum medium on Day 3 and the samples were fixed and stained for MyHC expression on Day 10. Culture medium was changed every other day. (c) To assess the influence of hydrogel formulation on myotube formation, we developed a simple screen using a fluorescence‐based plate reader assay to quantify MyHC staining. (d) The gel samples were cultured individually in 96‐well plates. A 30 × 30 raster scan (Excitation: 544/20 nm; Emission: 575/20 nm, Mirror: 560 nm) was performed on each well to measure the level of MyHC labelling in each sample. A 4 × 4 subset was selected in the gel disk region and a second 4 × 4 subset was selected in the background well region. After correcting for the blank control value, the difference between the mean gel and mean background was taken as the mean MyHC fluorescence for each gel. (e) The mean fluorescence values for each of the hydrogel formulations (*n* = 3–5), gray denotes that a sufficient number of samples were not obtained for this condition

We selected GelMA wt%, cell density, and Matrigel concentration as the test parameters for the parametric analysis (Figure 2a). As myoblasts are cultured normally on Matrigel‐coated TCP, we included Matrigel as a potential additive in the hydrogel formulations. Considering experimental constraints related to forming and handling GelMA disks, and our preliminary observations, we chose 3, 4, and 5 wt% GelMA for these experiments. The moduli of the 3, 4, and 5 wt% GelMA hydrogels were measured as *E* = 0.8, 3.7, and 8.0 kPa, respectively (Figure S2). Myotube formation requires fusion of myoblasts, and this process is often related to the confluence of myoblasts in 2D, which influences the probability of cell fusion. To investigate the role of cell density, we chose three cell densities (1.25, 2.5, and 5.0 × 10^6^ cells ml^−1^), such that it would be possible to observe the encapsulated cells and myotube formation using standard microscopy even at the highest density. Matrigel concentrations (0, 0.4, 0.8, and 1.2 mg ml^−1^ final concentration) were selected to prevent Matrigel from forming a gel on its own during sample preparation.

Hydrogel precursor solutions were prepared for each of the conditions in the parametric analysis and myoblasts were encapsulated in 10 μl hydrogel disks (*n* = 3–5; Day 0). After initiating differentiation on Day 3, the samples were observed for myotube formation and spontaneous myotube contraction for an additional 7 days (Figure 2b). The samples were then fixed and stained for myosin heavy chain (MyHC) expression, a standard marker of myotube formation, on Day 10. Assessing the influence of hydrogel composition on myotube formation and spontaneous myotube contraction with visual inspection can be subjective and quantifying the extent of myotube formation using confocal microscopy in a large number of gel samples can be time consuming and cost‐prohibitive. Furthermore, common methods like extraction of suitable protein or RNA for Western blotting or PCR is more challenging in 3D culture than in traditional 2D culture. In this study, we were not able to extract high quality protein or RNA from 3D GelMA samples for further downstream analysis by Western blotting or qPCR (data not shown). Therefore, we adapted a multi‐well plate reader to evaluate the fluorescence of all the encapsulation conditions as a rapid method to quantify the influence of hydrogel formulation on MyHC expression, taken as an indicator of myotube formation (Figure 2c,d).

In the plate reader analysis of the gel samples, we observed that MyHC staining (myotube formation) depended on the hydrogel formulation (Figure 2e). MyHC staining varied most significantly with the concentration of GelMA used to prepare the sample; MyHC increased with decreasing GelMA wt%. This observation was corroborated by bright‐field imaging of the various hydrogel formulations during the 10 day culture and more spontaneously contracting myotubes were observed in the 3 wt% GelMA samples (Figure S3). A few elongated myotubes were observed in 4 wt% GelMA samples, while the cells remained mostly rounded in the 5 wt% GelMA samples (Figures S4 and S5). We attributed this effect to the ability of myoblasts to spread and migrate more freely in lower concentration GelMA gels and, therefore, the cells were able to interact and fuse.

Contrary to our initial hypothesis, increased cell density did not correspond to increased MyHC staining (myotube formation). In contrast, MyHC staining correlated weakly with cell density. This may indicate that 1.25 × 10^6^ cells ml^−1^ was already suitable for efficient myotube formation or may be an artifact of the selected times for initiating differentiation (Day 3) and the end point of the experiment (Day 10). Further, nutrient limitations may influence cell behavior at high cell density in 3D culture.

The effect of Matrigel concentration was also more subtle. The effect was seen most clearly in 3 wt% GelMA samples with higher cell seeding densities (2.5 and 5 × 10^6^ cells ml^−1^) and in 4 wt% GelMA samples with low seeding density (1.25 × 10^6^ cells ml^−1^). In these three cases, MyHC staining (myotube formation) increased in conditions where Matrigel was included. However, there was not a strong effect across the range of concentrations of Matrigel that were tested (0.4, 0.8, and 1.2 mg ml^−1^). This suggested that the presence of Matrigel facilitated myotube formation, but that the amount of Matrigel (above 0.4 mg ml^−1^) did not have a considerable effect.

To analyze the statistical relevance of each of the variables on MyHC staining (myotube formation), we performed a three‐way ANOVA on the data obtained from the plate reader‐based screen. A complete description of the statistical analysis of the screen is included in the Supporting Information. GelMA wt%, Matrigel concentration, and cell density were used as independent variables with fluorescence readings of MyHC staining as the output. The *p*‐values for GelMA wt% (9.135 × 10^−8^), Matrigel concentration (0.102), and cell density (0.090) indicate that the primary influence on myotube formation was GelMA wt%. An additional ANOVA, treating Matrigel as a categorical variable instead of a continuous variable, demonstrated that the presence of Matrigel also had a significant effect (p = 0.011) on MyHC staining. Cell density did not have a significant effect on MyHC staining.

**FIGURE 3:**
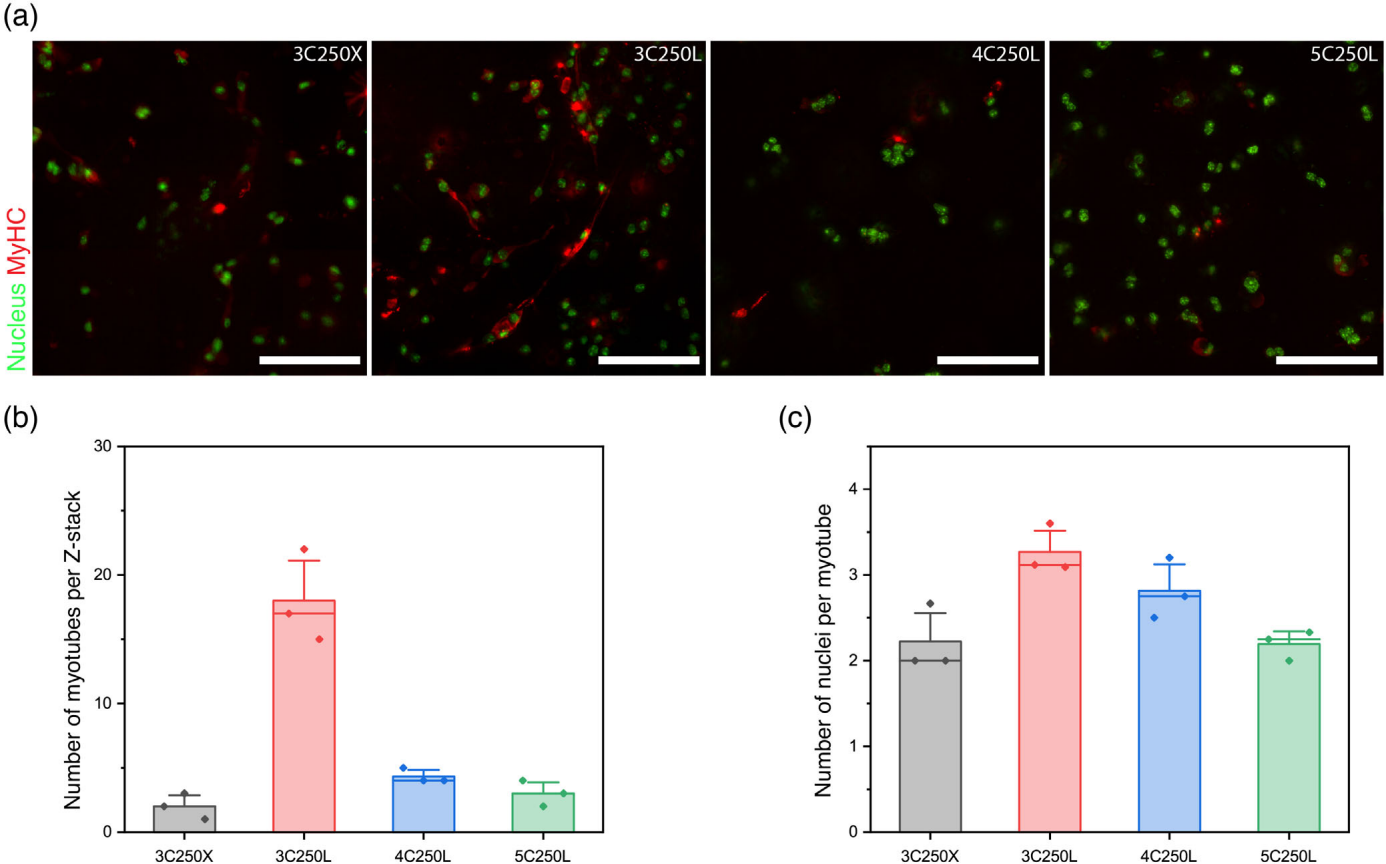
Image analysis for myotube formation in select hydrogel formulations. (a) Fluorescent micrograph of myotube formation in 3C250X: 3 wt% GelMA samples, 2.5 × 10^6^ cell ml^−1^ without Matrigel (0.0 mg ml^−1^) and 3C250L: with Matrigel (0.4 mg ml^−1^), 4C250L: 4 wt% GelMA sample, 2.5 × 10^6^ cell ml^−1^ with Matrigel (0.4 mg ml^−1^), 5C250L: 5 wt% GelMA sample, 2.5 × 10^6^ cell ml^−1^ with Matrigel (0.4 mg ml^−1^). Scale bars, 100 μm. (b) Number of myotubes per Z‐stack for the same four conditions. Fewer myotubes per stack were observed in 4 and 5 wt% GelMA samples as well as in samples without Matrigel. (c) Number of nuclei per myotube in Z‐stack for the same four conditions. The number of nuclei per myotube did not vary dramatically over these conditions with the highest number of nuclei per myotube observed in the 3C250L sample

To corroborate the findings from the screening based on MyHC staining, we also imaged select conditions for the presence of multinucleated myotubes (Figure 3). After staining for MyHC (red) and labelling the cell nuclei (green), an increased density of myotubes was observed in the 3 wt% GelMA with Matrigel (0.4 mg ml^−1^) samples (Figure 3a). We also captured full thickness Z‐stacks of the samples and analyzed these images (*n* = 3 per condition) for the number of myotubes per Z‐stack (Figure 3b) and the number of nuclei per myotube (Figure 3c). Both the number of myotubes per Z‐stack and the number of nuclei per myotube correlated with the data from the plate reader‐based screen, suggesting that 3 wt% GelMA with Matrigel (0.4 mg ml^−1^) led to improved myotube formation as compared with 3 wt% GelMA without Matrigel (0.0 mg ml^−1^) or 4 and 5 wt% GelMA with Matrigel (0.4 mg ml^−1^).

Finally, we also observed spontaneous contraction of myotubes at later stages (Day 10) of the culture time in select conditions without any external stimulus (Videos S1–S3). Myotube contraction was observed more frequently in samples with lower GelMA wt% that contained Matrigel. For 3 wt% GelMA samples with 1.25 × 10^6^ cells ml^−1^, myotube contraction was observed for all Matrigel concentrations (Video S1). In 3 wt% GelMA samples with higher cell densities, we observed myotube contraction in two conditions (2.5 × 10^6^ cells ml^−1^ with 0.4 mg ml^−1^ Matrigel and 5 × 10^6^ cells ml^−1^ with 0.8 mg ml^−1^ Matrigel; Videos S2 and S3, respectively). These observations should be considered representative and do not imply the inability to form contracting myotubes in all other conditions.

A core finding of this study is that experimental design and common plate reader‐based assays can be used to guide hydrogel formulation selection for 3D cell culture. Here, we found that myotube formation from primary myoblasts was favored in lower wt% GelMA hydrogels supplement with Matrigel. However, we did not identify the specific properties of these gels that facilitate or drive myogenesis in these samples. Lower GelMA wt% affects both the stiffness of the network as well as the bioactive ligand density. The presence of Matrigel can also affect matrix stiffness, porosity, and bioactive ligand density. Therefore, additional controlled studies should be carried out to isolate the specific signals that encourage myogenesis in 3D hydrogels. Understanding the specific extracellular effects that drive myogenesis could further guide the development of biomaterials for skeletal muscle repair and the design of in vitro cellular models.

## CONCLUSION

In total, these findings suggest that low wt% GelMA hydrogels with Matrigel as an additive comprise suitable hydrogel formulations to facilitate myotube formation from primary murine myoblasts in 3D. Further, we demonstrated that commonly available fluorescence‐based plate readers can be used to develop rapid screening methods to assay 3D hydrogel formulations based on the expression and staining for a known marker of the desired functional output. This approach complements standard analysis via high content imaging or molecular biology, which can be challenging and slow in 3D samples, especially when the sample number is large.

## MATERIALS AND METHODS

All materials were purchased from Sigma‐Aldrich unless stated otherwise.

### Preparation and characterization of methacryloyl gelatin

Gelatin was functionalized with methacrylate moieties following previously published protocols.^18^, ^19^, ^20^ Briefly, methacrylic anhydride (12 g; 276685) was added to gelatin (20 g; 300 bloom, type A; G2500‐100G) dissolved in deionized water (50°C) and allowed to react for 120 min. The reaction solution was then cooled to room temperature and unreacted methacrylic anhydride was removed by centrifugation (3,500 RCF for 5 min). The supernatant was dialyzed (SnakeSkin TM dialysis tubing, 3.5 MWCO, Ref 88244, Thermo Scientific) and lyophilized. The degree of functionalization of the obtained powder was characterized via NMR spectroscopy and determined to be ~45% (Figure S7).

### Cell culture and encapsulation

Primary murine myoblasts were isolated from C57BL/6J mice following established protocols.^21^ The mice were housed at ETH Zürich, tissue collection was performed under a protocol approved by the Zürich Cantonal Veterinarians office (ZH108/18). Tissue culture polystyrene dishes (*Ø* = 150 mm) were coated with Matrigel (diluted 1:25 in low glucose DMEM, Thermofisher 31885023) to improve cell attachment. Myoblasts were cultured on Matrigel‐coated dishes in a medium containing equal parts DMEM (Thermofisher 41966029) and F‐10 medium (Thermofisher 22390025) supplemented with 10% horse serum (Thermofisher 16050122), 20% fetal bovine serum (FBS; Thermofisher 10270106), 1% penicillin‐streptomycin (PenStrep; Gibco, 15140‐122), and 10 ng ml^−1^ basic fibroblast growth factor (bFGF; Bio‐Techne 233‐FB‐500) in a 5% CO_2_ incubator at 37°C.^22^ Passage 12 myoblasts were trypsinized and suspended in the hydrogel precursor solutions, consisting of specific concentrations of GelMA and Matrigel. The cell density of the final solution was varied according to the specified parameters. Lithium phenyl‐2,4,6‐trimethylbenzoylphosphinate (LAP; 0.1 wt%) was used as a photoinitiator for hydrogel polymerization and cell encapsulation under UV light (*λ* = 365 nm; *I* = 20 mW cm^−2^; *t* = 30 s). 10 μL of hydrogel precursor solution with cells were polymerized between two Sigmacote‐coated glass plates separated by a 1 mm silicone spacer to prepare each gel sample. The hydrogel disks were removed carefully from the glass surface and transferred immediately to culture medium. The medium was refreshed 30 min after gel formation.

Differentiation was induced in all encapsulated cells 3 days after encapsulation by changing to a low‐serum culture medium: knockout‐DMEM (KO‐DMEM; Thermofisher 10829018) supplemented with 1% MEM‐NEAA (Thermofisher 11140035), 1% GlutaMax (Thermofisher 35050038), 1% PenStrep (ThermoFisher, 15140122), 0.1% beta‐mercaptoethanol (Thermofisher 21985023), and 2% horse serum (Thermofisher 16050122). Gels were incubated in the differentiation medium for 7 days and the medium was refreshed every other day.

### Design of experiments for screening hydrogel encapsulation conditions

We chose three parameters in the hydrogel formulation (GelMA wt%, cell density, and Matrigel concentration) to vary and investigate their effect on myotube formation. We assessed the extent of myotube formation via immunostaining for myosin heavy chain (MyHC) 7 days after initiating differentiation. Other parameters such as time at which differentiation was initiated and concentration of photoinitiator were not considered in this work. After initial experiments and considering practical constraints, we chose three weight percentage conditions (3, 4, and 5 wt% of GelMA); three cell encapsulation densities (1.25, 2.5, and 5 × 10^6^ cells ml^−1^); and four Matrigel concentrations (0, 0.4, 0.8 and 1.2 mg ml^−1^). Using these parameters, we encapsulated myoblasts in a total of 36 hydrogel compositions. All conditions were encapsulated using the protocol described above.

### Immunostaining and plate reader screening

After incubation in differentiation medium for 7 days, the cells were washed twice in DPBS +/+ for 5 min at room temperature. The samples were then fixed in 4% paraformaldehyde solution (in DPBS +/+) for 45 min at room temperature. Following this, the samples were washed twice in DPBS +/+ for 5 min each and incubated in a blocking solution (2% donkey serum and 0.5% triton X‐100 in PBS) for 1 hr at room temperature. The samples were stained for MyHC by incubation first in the primary antibody solution (1 μg ml^−1^ mouse anti‐myosin heavy chain monoclonal antibody in blocking solution; Biotechne MAB4470‐SP) overnight at 4°C. The samples were then washed (0.5% Tween 20 in PBS) for 10 min at room temperature and then incubated in the secondary antibody solution (2.5 μg ml^−1^ Donkey Anti‐Mouse IgG NorthernLights™ NL557‐conjugate; Biotechne, NL007) overnight at 4°C. The samples were then washed twice with PBS for 5 min at room temperature.

The level of MyHC staining in the hydrogel samples was then screened with a fluorescence‐based plate reader assay (Hidex Sense). To measure the fluorescence signal in the hydrogel disks, we performed a 30 × 30 raster scan of each well in the 96‐well plate (Excitation: 544/20 nm; Emission: 575/20 nm). From each raster scan, two areas (4 × 4) were chosen (area of interest near the center of the gel and the background from the well and surrounding medium). The fluorescence value of each measurement point in these areas were averaged and the difference of these two values (center of gel less the background) was taken as the fluorescence value for that particular hydrogel sample (Figure S6). Similarly, a value was assigned for the blank gels (same hydrogel composition but without cells). This value was then subtracted from the fluorescence value of the corresponding hydrogel sample will cells to calculate the representative MyHC fluorescence.

### Microcopy

Contracting myotubes that formed within the samples were imaged with an inverted microscope (Nikon Eclipse Ts2; 10×/0.30 Plan Fluor objective equipped with an iDS camera). Bright‐field images during culture were obtained with an inverted microscope (EVOS M5000, Invitrogen; 20× objective). For immunofluorescence imaging, cell nuclei were stained (Biotracker 488 Green Nuclear Dye, SCT120; 1X in PBS for 60 min) after staining for MyHC to visualize the multinucleated myotubes. Fluorescent microscopy was performed on a Leica THUNDER 3D Imaging system (40×/0.60 FLUOTAR L objective) using the following THUNDER Large Volume Computational Clearing settings (40×/0.6 objective), Feature Scale (nm): 4,194, Strength (%): 92, Adaptive Deconvolution set with a refractive index of the mounting medium of 1.33.

### Statistical analysis

Three‐way ANOVA was conducted to analyze the results obtained from the fluorescence‐based plate reader assay. GelMA wt%, cell density, and Matrigel concentration were chosen as independent continuous variables. Another ANOVA was conducted considering Matrigel concentration as a categorical variable. A MATLAB code was used to calculate the influence of these factors on the plate reader values. More details about the statistical analysis can be found in Supporting Information.

## CONFLICT OF INTEREST

The authors declare no competing financial interests.

### AUTHOR CONTRIBUTIONS

Dhananjay V. Deshmukh and Mark W. Tibbitt wrote the manuscript. Dhananjay V. Deshmukh, Nils Pasquero, Gajraj Rathore, Joel Zvick, Ori Bar‐Nur, Jurg Dual, and Mark W. Tibbitt designed the experiments and interpreted the results. Dhananjay V. Deshmukh, Nils Pasquero, Gajraj Rathore, and Joel Zvick performed the experiments and analyzed the data. Joel Zvick and Ori Bar‐Nur provided the myogenic cells and supported the cell culture experiments. All authors reviewed and approved the final manuscript.

## Supporting information


**Appendix** S1: Supporting Information
