# Supplementary material for: Screening method to identify hydrogel formulations that facilitate myotube formation from encapsulated primary myoblasts
Source: Bioeng Transl Med. 2020 Sep 3;5(3):e10181. doi: 10.1002/btm2.10181 (PMC7510461; doi:10.1002/btm2.10181)
Supplement: Supplementary file 1 — Appendix S1: Supporting Information [file BTM2-5-e10181-s001.zip › btm210181-sup-0001-supinfo.docx]

**Supporting Information**

**Screening method to identify hydrogel formulations that facilitate myotube formation from encapsulated primary myoblasts**

Dhananjay V. Deshmukh, Nils Pasquero, Gajraj Rathore, Joel Zvick, Ori Bar-Nur, Jurg Dual, and Mark W. Tibbitt^*^

*Corresponding author: [mtibbitt@ethz.ch](mailto:mtibbitt@ethz.ch)

**Table of Contents**

**Supporting Figure S1 Preliminary experiments……………………………………...3**

**Supporting Figure S2 Rheological measurements for GelMA………………………5**

**Supporting Figure S3 Bright field images of 3 wt% samples………………………..6**

**Supporting Figure S4 Bright field images of 5 wt% samples………………………..6**

**Supporting Figure S5 Bright field images of 5 wt% samples………………………..7**

**Supporting Figure S6 Calculation of fluorescence values…..………………………..8**

**Supporting Figure S7 Proof-of-concept experiment for plate reader…………….....9**

**Supporting Methods Statistical analysis.…………………..………………….........11**

**Supporting Table S1 & S2 ANOVA analysis results……………………………………..12**

**Supporting Figure S8 NMR characterization of methacryol gelatin.………...……13**

**Supporting Video 1-3 Spontaneously contracting myotubes……………………….14**

**Supporting Video 4-7 Z-Stack of entire thickness of hydrogel…………………….14**

**Supporting Video 8 Non-identifiable contractile cell……………………………..14**

**Preliminary myoblast encapsulation experiments with peptide-functionalized, poly(ethylene glycol)-norbornene (PEG-MMP) and GelMA hydrogels.**


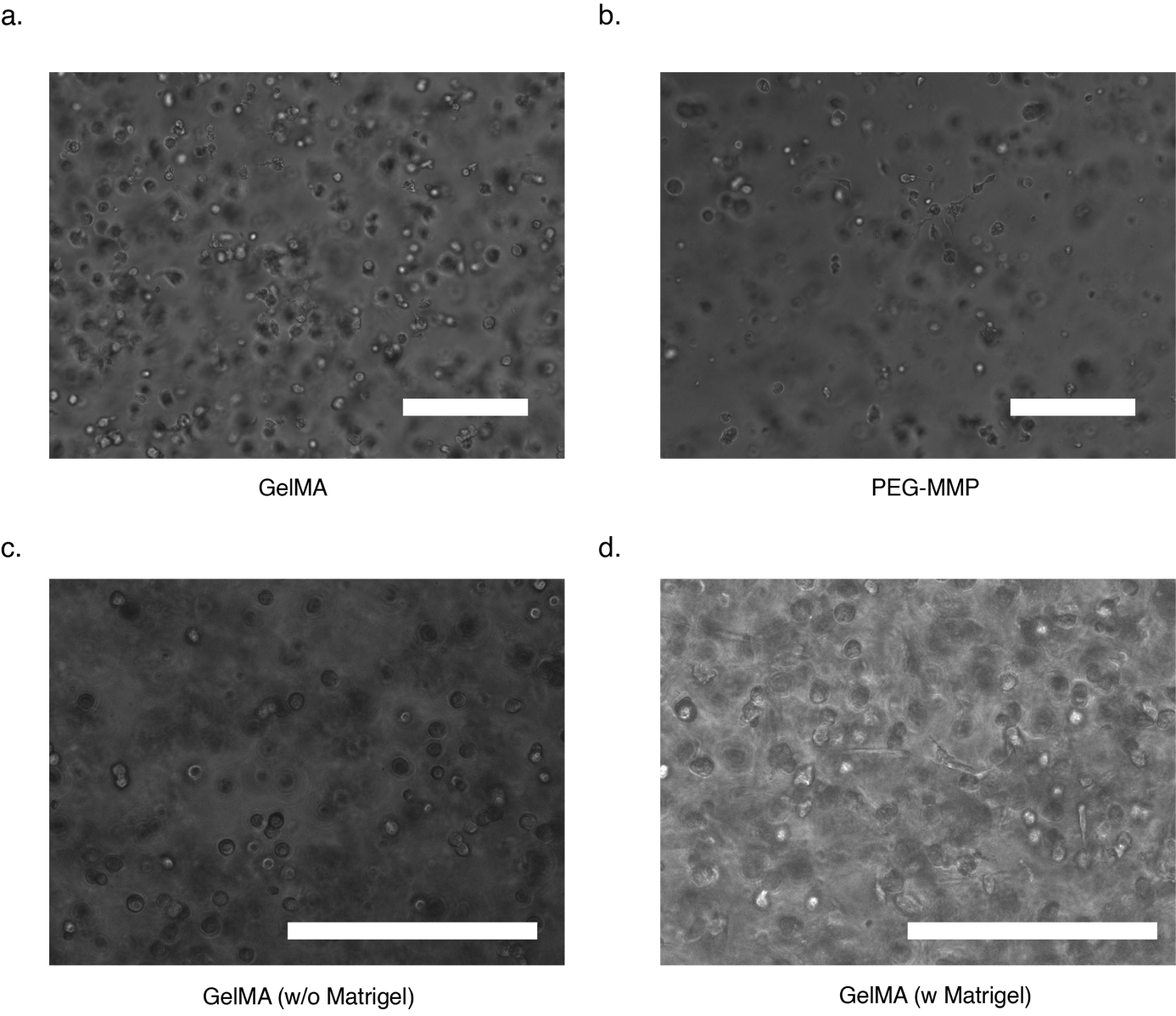


**Figure S1.** Myoblasts encapsulated in (a) 5 wt% GelMA, 8 days post encapsulation, (b) 4 wt% PEG-MMP, 8 days post encapsulation, (c) 3 wt% GelMA, without Matrigel, 5 days after initiating differentiation, (d) 3 wt% GelMA, with 1.2 mg mL^-1^ Matrigel, 5 days after initiating differentiation.

In preliminary experiments, we encapsulated primary myoblasts in GelMA and PEG-MMP hydrogels. For the PEG-MMP hydrogels, we prepared gels using 4 wt% poly(ethylene glycol) (PEG; 4ARM, M_n_ ~20 kDa) end-functionalized with norbornene moieties cross-linked with matrix metalloproteinase (MMP) cleavable peptides (6.1 mM, 0.47 wt%; KCGPQGIWGQCK, Genscript) and RGD for cell adhesion (1.5 mM; CRGDS, Bachem). The amount of MMP peptide is calculated in such a way that the moles of MMP are given as,

n_MMP_ = (n__NORBORNENE_ - n_RGD_)/2

to preserve 1:1 stoichiometry between thiols and norbornenes.

All solutions were prepared in Tris buffer. Myoblasts expanded on Matrigel-coated petri dishes were resuspended in 4 wt% PEG-MMP and 5 wt% GelMA solutions (1 x 10^6^ cells mL^-1^) and the gels were prepared via photopolymerization. After 8 days of culture in growth and differentiation media, we observed that myoblasts spread better in GelMA compared with PEG-MMP samples (Figure S1 a,b). Therefore, we decided to use GelMA for our screening experiments.

As myotube formation was not observed in the 5 wt% GelMA samples, we also prepared preliminary experiments in 3 wt% GelMA. After differentiation was initiated, limited myotube formation was also observed in 3 wt% GelMA (Figure S1 c). However, increased myotube formation was seen in GelMA supplemented with 1.2 mg mL^-1^ Matrigel (Figure S1 d), both 5 days after initiating differentiation. Scale bars, 300 μm.

**Rheological measurements of GelMA hydrogels.**


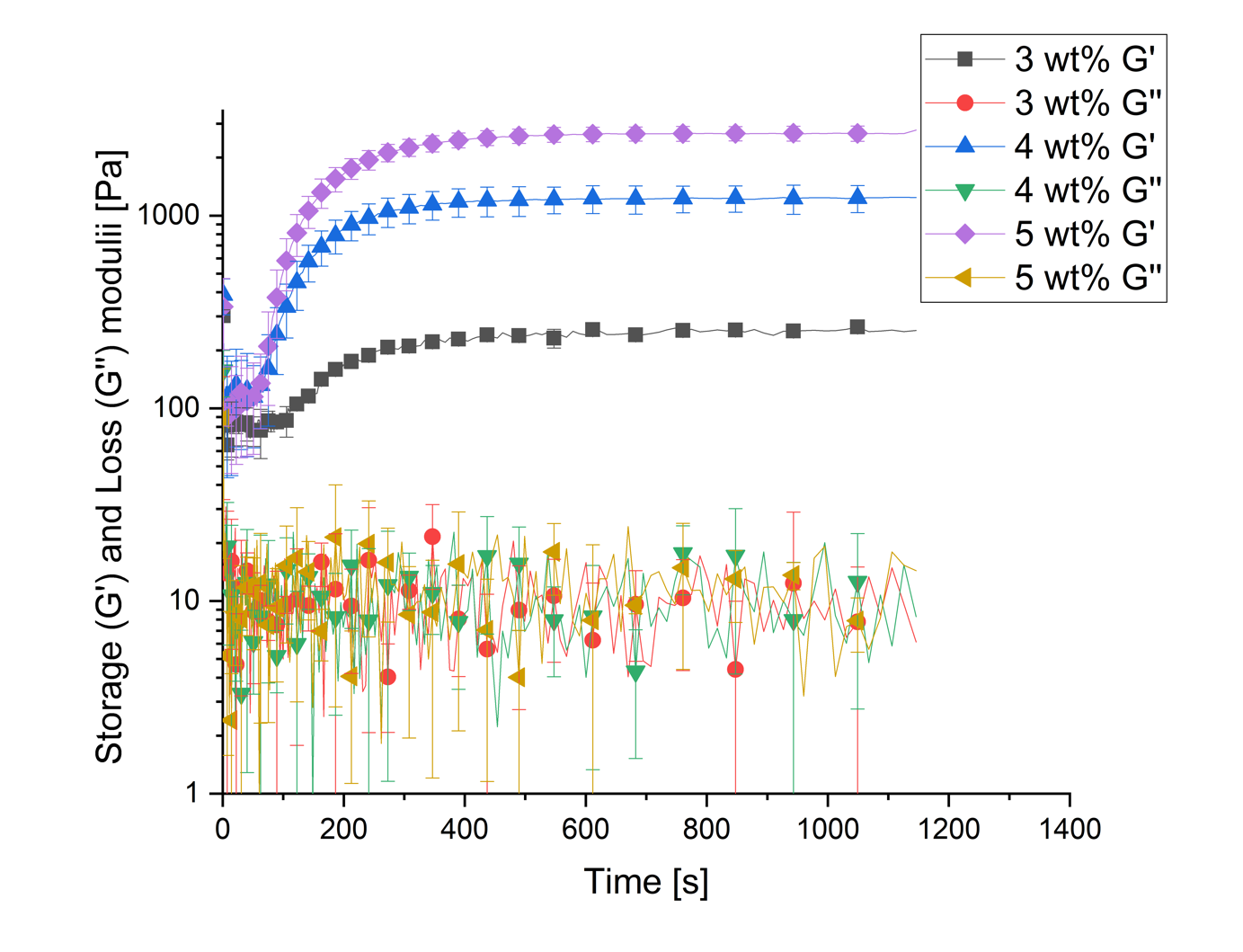
**Figure S2**. The shear modulus of GelMA hydrogels (3, 4, and 5 wt%) was measured using a shear rheometer (MCR 502; Anton Paar) equipped with a glass bottom plate for UV photopolymerization (λ = 365 nm, I = 2 mW cm^-2^) was used. The UV light was switched on at *t* = 30s. The shear storage (G') and loss (G'') moduli were measured during a time sweep using a sand-blasted parallel plate geometry (Ø = 8 mm; PP08/S, Anton Paar) at 10 rad s^-1^ oscillation frequency and 0.1% shear strain.

**Bright field images of samples.
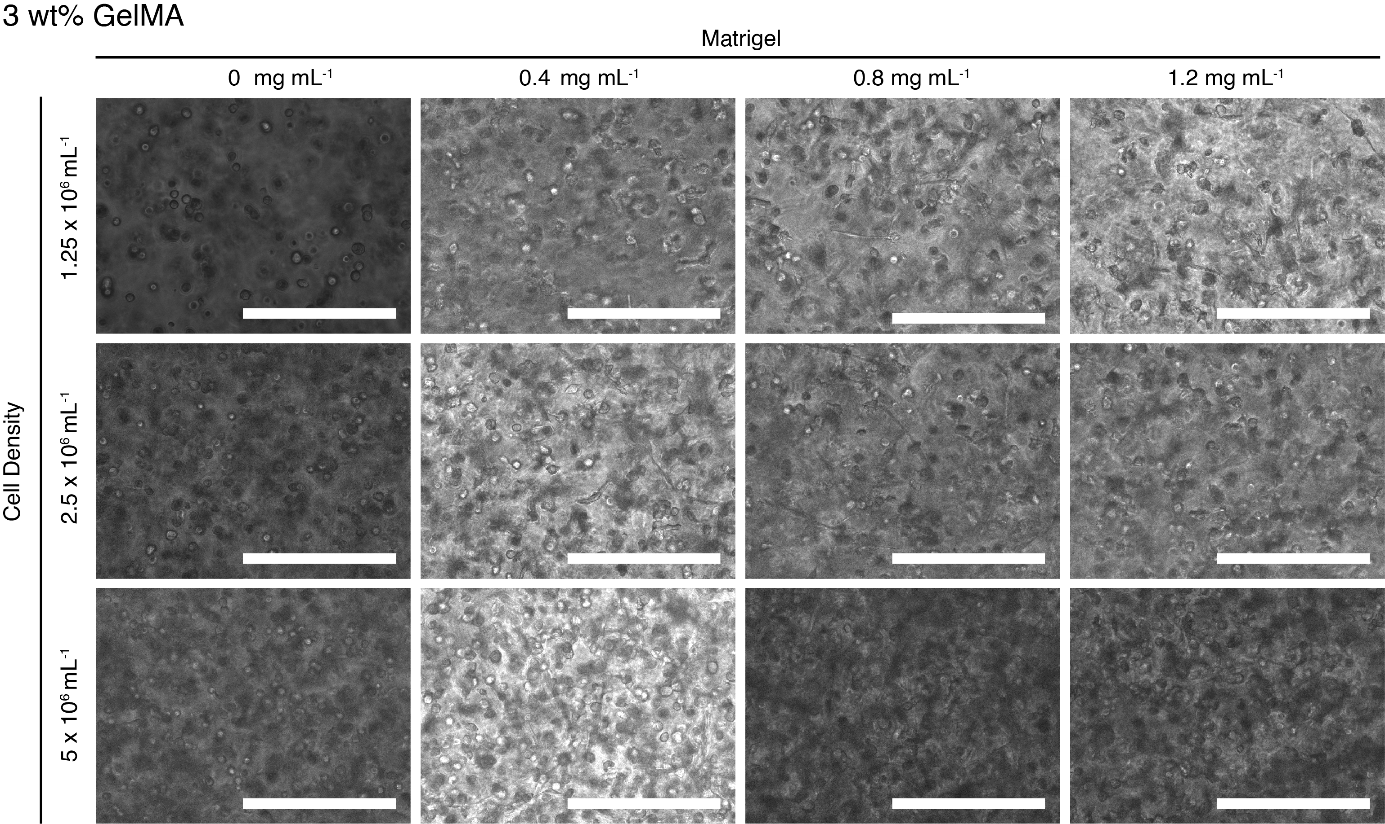
Figure S3:** Samples with 3 wt % GelMA, 5 days after initiating differentiation. **
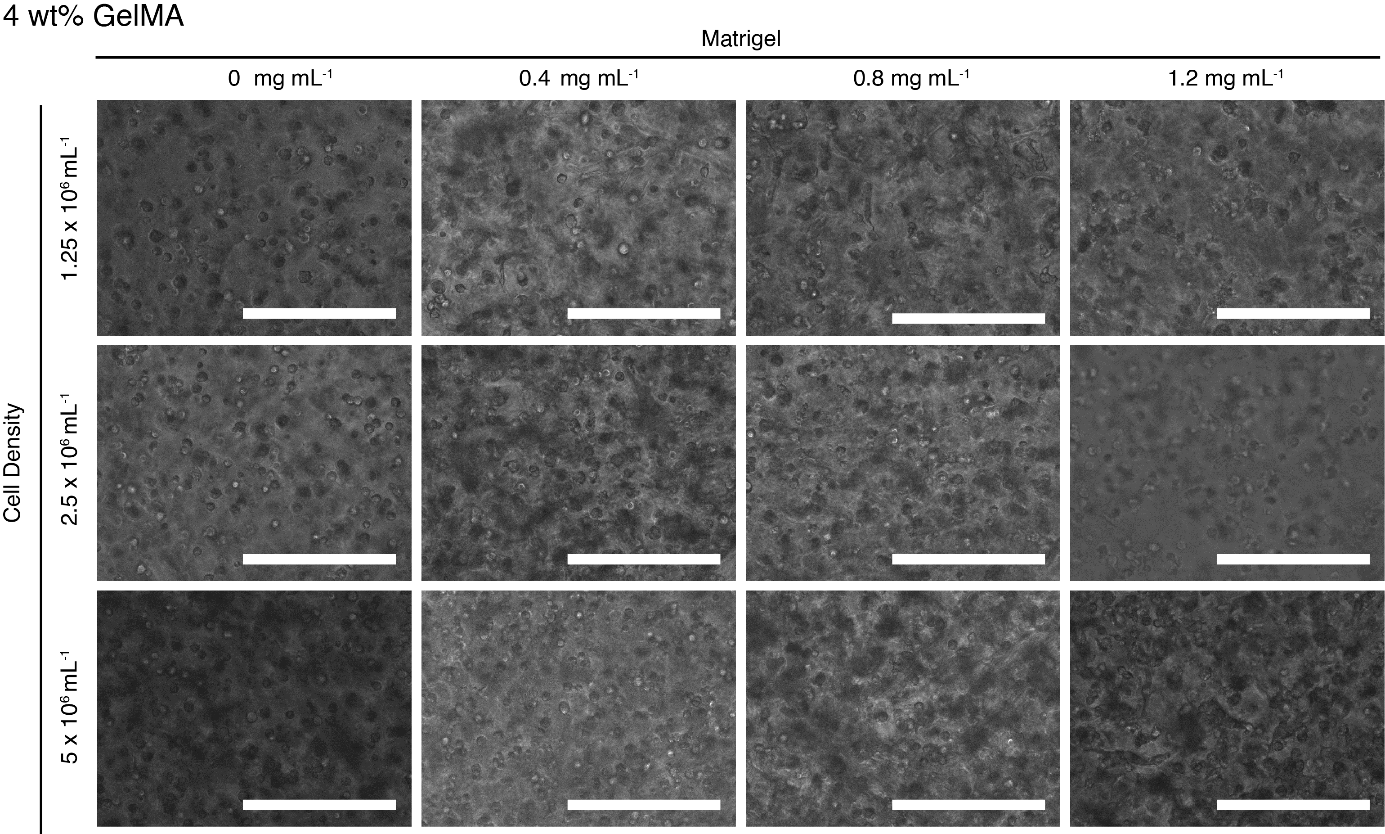
Figure S4:** Samples with 4 wt % GelMA, 5 days after initiating differentiation.


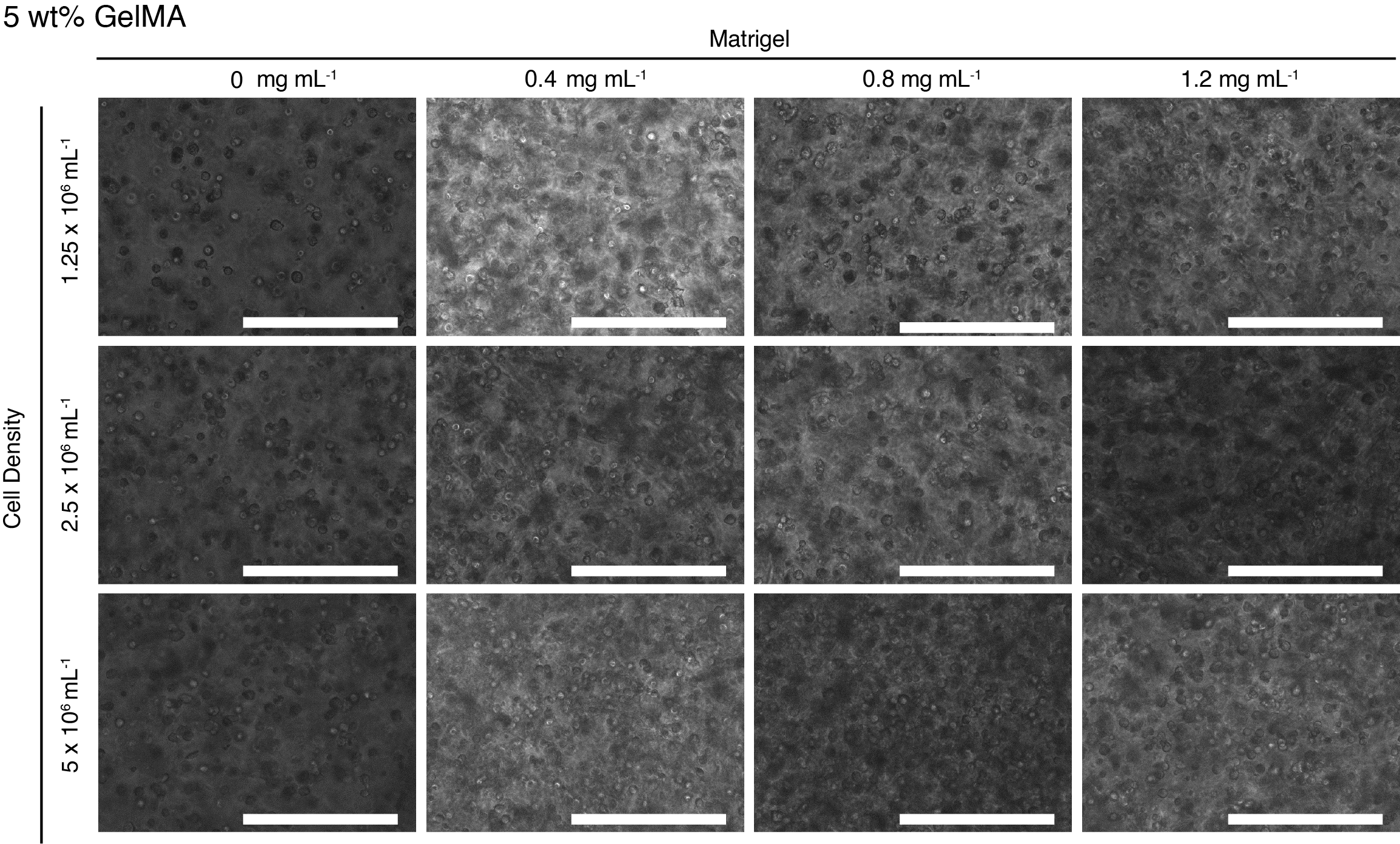


**Figure S5.** Samples with 5 wt % GelMA, 5 days after initiating differentiation.

**Calculation of representative fluorescence values.**

**
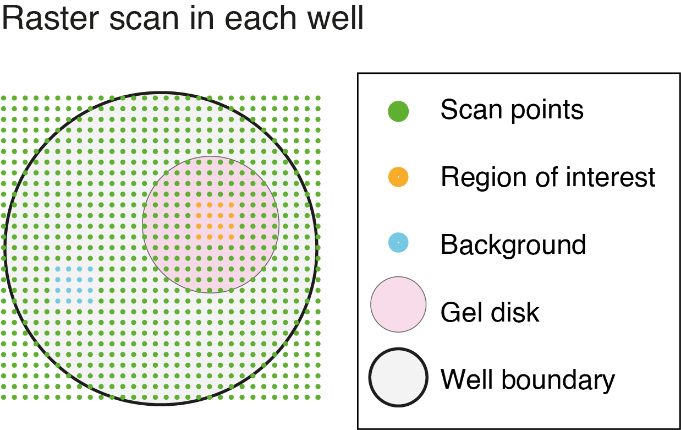
**

**Figure S6**. For each well, two sub-regions (4x4) were selected: orange scan points represent the area of interest near the center of the gel (A_gel_) and the blue points represent the area inside the well but outside the gel (A_bkg_; Figure S3). The fluorescence value was calculated for all gels that appeared complete and were not tilted within the wells. This fluorescence value was calculated by subtracting the mean fluorescence value of points in A_bkg_ from the mean fluorescence value of points in A_gel_, denoted by F_bkg_ and F_gel_, respectively. Therefore, the fluorescence of the well was written as,

F_well_ = F_gel_ - F_bkg_

To get the fluorescence value representative of the MyHC present in the samples, F_well_ of similar samples (same GelMA wt% and Matrigel concentration) but without cells was subtracted from F_well_ of the corresponding samples with cells.

**Proof-of-concept analysis for 3D fluorescence of plate reader**

NIH 3T3 fibroblasts were cultured on dishes in high glucose DMEM (Thermofisher 31966021) supplemented with 10% fetal bovine serum (FBS; Thermofisher 10270106), and 1% penicillin-streptomycin (PenStrep; Gibco, 15140-122). P6 fibroblasts were encapsulated in 4 wt% GelMA without any Matrigel (0.0 mg mL^-1^). 0.1 wt% LAP was used as a photoinitiator for hydrogel polymerization and cell encapsulation under UV light (λ = 365 nm; *I* =20 mW cm^-2^; *t* = 30 s). Five hydrogel precursor solutions with variable cell densities (1.0, 2.0, 3.0, 4.0, and 5.0 x 10^6^ cells mL^-1^) were prepared along with a blank sample containing no cells. 10 μL of the solution polymerized between two Sigmacote-coated glass plates separated by a 1 mm silicone spacer to prepare each gel sample. The hydrogel disks were removed carefully from the glass surface and transferred immediately to culture medium. After 2 h in the cell media, the samples were washed and fixed similar to the other gel samples in this work. The cell nuclei were stained with Biotracker 488 Green Nuclear Dye (SCT120) according to manufacturer’s instructions. The samples were washed twice with PBS before screening with fluorescent plate reader.

At least 5 gels were analyzed per condition using a method similar to described in the previous section. To measure the fluorescence signal of the nuclear dye in the hydrogel disks, we performed a 30x30 raster scan of each well in the 96-well plate (Excitation: 485/10 nm; Emission: 535/20 nm, Mirror: 505 nm). The obtained data was processed in the same manner as the developed plate reader-based screening method. We observed a linear correlation between cell density and fluorescent signal up to ~4.0 x 10^6^ cells mL^-1^. (Figure S7) Beyond this density the signal saturates. However, since the intensity obtained for MyHC^+^ staining is significantly lower than the nuclei staining, the instrument should be capable of detecting fluorescence signal corresponding to MyHC staining with our proposed method.


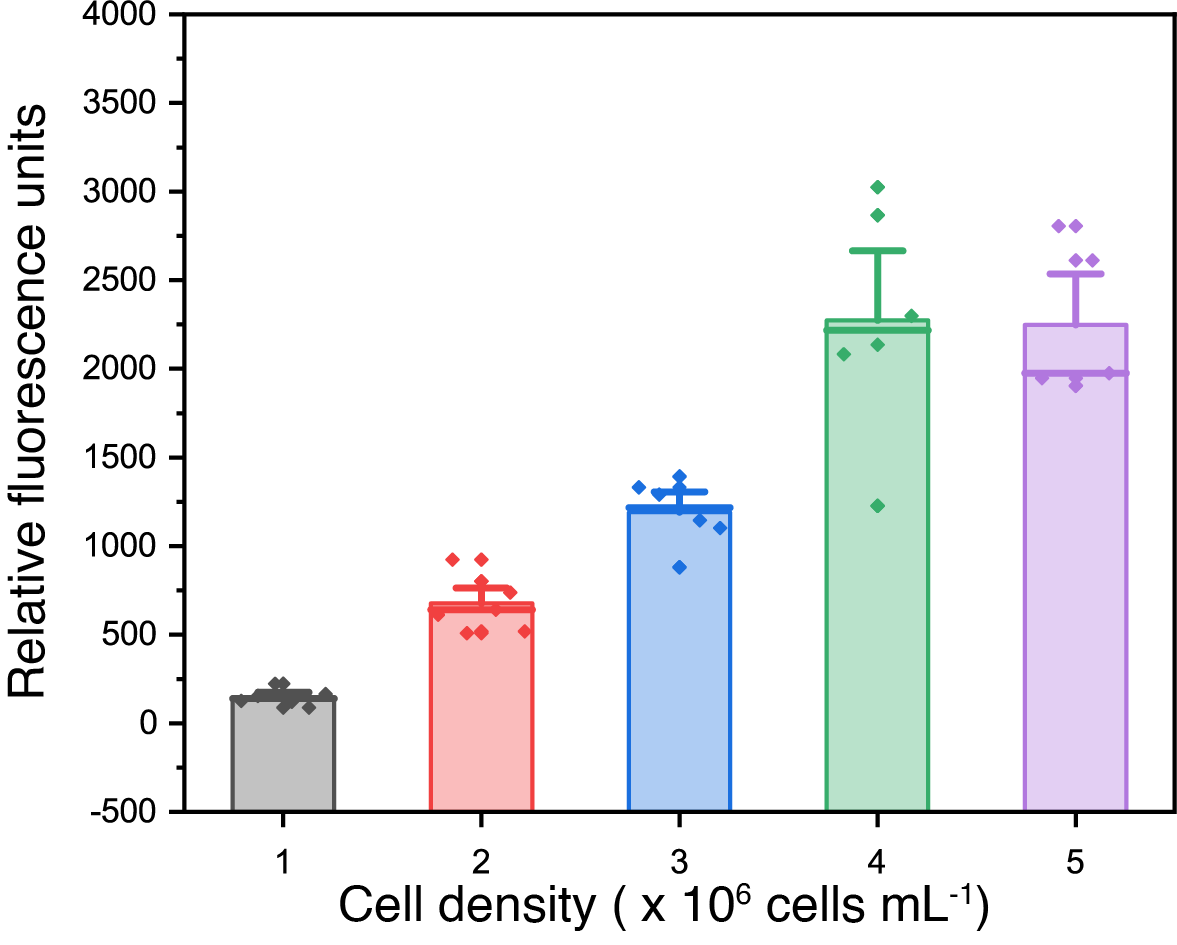


**Figure S7.** NIH 3T3 fibroblasts were encapsulated in 4 wt% GelMA hydrogels using LAP as the photoinitiator (0.1 wt%) under UV light (λ = 365 nm; I =20 mW cm^-2^; *t* = 30 s) at cell densities of 1.0, 2.0, 3.0, 4.0, or 5.0 x 10^6^ cells mL^-1^. The samples were cultured for 2 h and then fixed in 4% PFA as described in section 4.4. The samples were stained with a nuclear dye (1X Biotracker 488 green nuclear dye; Merck SCT120) for 60 min at RT, and then rinsed twice with PBS. To measure the fluorescence signal of the nuclear dye in the hydrogel disks, we performed a 30x30 raster scan of each well in the 96-well plate (Excitation: 485/10 nm; Emission: 535/20 nm, Mirror: 505 nm). The obtained data was processed in the same manner as the developed plate reader-based screening method*.*

**Statistical Analysis**

We conducted a three-way ANOVA on the results obtained from the fluorescence-based plate reader assay. The three independent variables were GelMA wt%, cell density, and Matrigel concentration. The three groups were compared against the output which was recorded as the adjusted fluorescence intensity.

ANOVA table (Table S1) was been generated using a MATLAB code based on the following data:

| Gel wt% | Cell density | Matrigel | Output |
| --- | --- | --- | --- |
| 4 | 125 | 0 | 72.23438 |
| 5 | 125 | 0 | 28.025 |
| 3 | 125 | 0.4 | 163.4208 |
| 4 | 125 | 0.4 | 111.2656 |
| 5 | 125 | 0.4 | 84.275 |
| 3 | 125 | 0.8 | 167.9667 |
| 4 | 125 | 0.8 | 120.1469 |
| 5 | 125 | 0.8 | 23.84167 |
| 3 | 125 | 1.2 | 155.6875 |
| 4 | 125 | 1.2 | 101.8594 |
| 5 | 125 | 1.2 | 50.90104 |
| 3 | 250 | 0 | 71.21875 |
| 4 | 250 | 0 | 42.85938 |
| 5 | 250 | 0 | 42.0375 |
| 3 | 250 | 0.4 | 110.5958 |
| 4 | 250 | 0.4 | 52.9375 |
| 5 | 250 | 0.4 | 49.1875 |
| 3 | 250 | 0.8 | 107.1875 |
| 4 | 250 | 0.8 | 45.42188 |
| 5 | 250 | 0.8 | 55.92917 |
| 3 | 250 | 1.2 | 123.5313 |
| 4 | 250 | 1.2 | 49.67188 |
| 5 | 250 | 1.2 | 29.14167 |
| 3 | 500 | 0 | 56.10938 |
| 4 | 500 | 0 | 49.51563 |
| 5 | 500 | 0 | 61.6875 |
| 3 | 500 | 0.4 | 145.2917 |
| 4 | 500 | 0.4 | 82.20833 |
| 5 | 500 | 0.4 | 2.325 |
| 3 | 500 | 0.8 | 136.9167 |
| 4 | 500 | 0.8 | 72.12188 |
| 5 | 500 | 0.8 | 45.24167 |
| 3 | 500 | 1.2 | 111.25 |
| 4 | 500 | 1.2 | 75.17188 |
| 5 | 500 | 1.2 | 60.34167 |

We compared the means of the three independent variables and analyzed their effect on fluorescence. Before conducting the analysis, we defined our null hypothesis. The type 1 error rate is set as 5% and the results are interpreted accordingly.

Null hypothesis: There is no effect of GelMA wt%, cell density, and Matrigel concentration on adjusted fluorescence intensity and they are all independent of each other.

|  | Df | Mean square | F value | Pr(>F) |
| --- | --- | --- | --- | --- |
| Cell density | 1 | 2209 | 3.06 | 0.090 |
| Gel wt% | 1 | 34579.73 | 47.94 | 9.13 x 10^-8^*** |
| Matrigel | 1 | 2045.05 | 2.83 | 0.102 |

**Table S1:** ANOVA analysis with all variables considered continuous, and described as rows. The columns represent the statistics, the last column signifying whether the variable is significant or not.

Significant codes: 0 ‘***’, 0.001 ‘**’, 0.01 ‘*’, 0.05 ‘.’.

From the ANOVA results, we found that GelMA wt% was the most influential factor on fluorescence intensity. Hence, we reject the Null hypothesis and concluded that GelMA wt% influenced MyHC expression. However, the other two variables did not influence fluorescence intensity significantly based on this analysis. The variance with Matrigel concentration could be low since samples with Matrigel did not show significant variations among themselves. To further investigate the effect of Matrigel, we carried out another ANOVA analysis with Matrigel concentration chosen as a categorical variable, with presence or absence of Matrigel being the two conditions. All Matrigel conditions where concentration is 0.4, 0.8, or 1.2 mg mL^-1^ were pooled together as ‘Matrigel present’ conditions and 0.0 mg mL^-1^ conditions were considered as ‘Matrigel absent’. The results for this analysis showed that presence or absence of Matrigel can indeed lead to significance variance. (Table S2)

|  | Df | Mean square | F value | Pr(>F) |
| --- | --- | --- | --- | --- |
| Cell density | 1 | 2020.93 | 3.17 | 0.090 |
| Gel wt% | 1 | 33668.14 | 52.92 | 3.47 x 10^-8^*** |
| Matrigel | 1 | 4684.44 | 7.36 | 0.010 . |

**Table S2:** ANOVA analysis with Matrigel concentration as categorical variable, the independent variables are described as rows. The columns represent the statistics, the last column signifying whether the variable is significant or not.
Significant codes: 0 ‘***’, 0.001 ‘**’, 0.01 ‘*’, 0.05 ‘.’.

**NMR characterization of methacryol gelatin.**


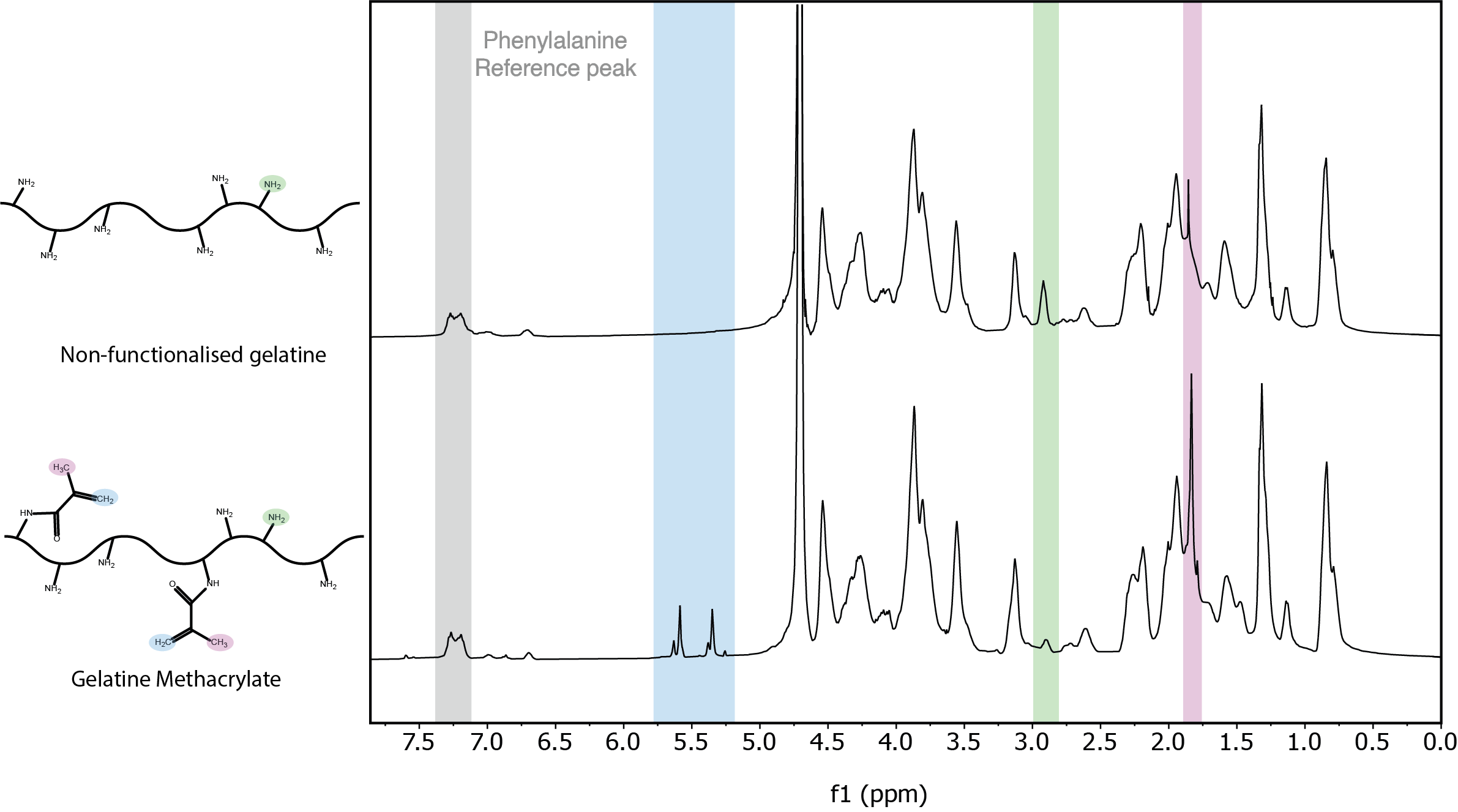


**Figure S8.** NMR spectra of unmodified gelatin (top) and methacryol gelatin (bottom) in deuterated H_2_O. Phenylalanine (7.1–7.4 ppm) was used to normalize the spectra. Following functionalization, gelatin lysine groups (2.8–3.0 ppm) were replaced by methacrylate moieties. The degree of functionalization was calculated as 100*(1-A(lysine of GelMA)/A(lysine Gelatin). The DoF for the GelMA used in this work was ~45%.

**Supplementary Videos**

Spontaneous contractility of formed myotubes within GelMA hydrogels.

**Supporting Video 1.** 3 wt% GelMA, 1.25 x 10^6^ cells mL^-1^, Matrigel = 0.4, 0.8, and 1.2 mg mL^-1^.

**Supporting Video 2.** 3 wt% GelMA, 2.5 x 10^6^ cells mL^-1^ with 0.4 mg mL^-1^ Matrigel.

**Supporting Video 3.** 3 wt% GelMA, 5 x 10^6^ cells mL^-1^ with 0.8 mg mL^-1^ Matrigel.

**Supporting Video 4.** Z-stack of the entire thickness of 3 wt% GelMA, 2.5 x 10^6^ cells mL^-1^, 0.0 mg mL^-1^ Matrigel

**Supporting Video 5.** Z-stack of the entire thickness of 3 wt% GelMA, 2.5 x 10^6^ cells mL^-1^, 0.4 mg mL^-1^ Matrigel

**Supporting Video 6.** Z-stack of the entire thickness of 4 wt% GelMA, 2.5 x 10^6^ cells mL^-1^, 0.4 mg mL^-1^ Matrigel

**Supporting Video 7.** Z-stack of the entire thickness of 5 wt% GelMA, 2.5 x 10^6^ cells mL^-1^, 0.4 mg mL^-1^ Matrigel

**Supporting Video 8.** A non-identifiable contractile cell in 3 wt% GelMA, 1.25 x 10^6^ cells mL^-1^, 0.4 mg mL^-1^ Matrigel
